# Patient Safety and Quality Improvement in Nursing Practice: Associations Among Workload, Occupational Coping Self-Efficacy and Medical Device-Related Pressure Injury Prevention

**DOI:** 10.3390/healthcare14020270

**Published:** 2026-01-21

**Authors:** Hyun Suk Gwag, Jin Ah Kim

**Affiliations:** College of Nursing, Dongguk University WISE Campus, Dongdaero 123, Gyeongju-si 38066, Republic of Korea; gwaghyunsuk0125@gmail.com

**Keywords:** medical device-related pressure injury (MDRPI), occupational coping self-efficacy, mediation analysis, preventive nursing practice, workload

## Abstract

**Background/Objectives**: Medical device-related pressure injury (MDRPI) is a significant patient safety issue associated with increased morbidity, prolonged hospitalization, and healthcare costs. Although evidence-based guidelines for MDRPI prevention exist, nurses’ prevention performance remains suboptimal, and the mechanisms linking workload to preventive practice remain insufficiently elucidated. Within a patient safety and quality improvement framework, this study aimed to examine whether occupational coping self-efficacy (OCSE) is statistically consistent with an indirect association linking nurses’ workload and MDRPI prevention performance across the nursing practice continuum. **Methods**: This descriptive correlational study used a mediation model with data from 181 registered nurses working in intensive care units, general wards, and integrated nursing care wards in South Korea. Workload, OCSE, and MDRPI prevention performance were measured using validated instruments. Mediation was tested using hierarchical regression and bootstrapped analysis (PROCESS macro Model 4, 5000 resamples), controlling for demographic and work-related variables. **Results**: Higher workload was associated with lower OCSE, while higher OCSE was associated with better MDRPI prevention performance. When OCSE was included in the model, the direct association between workload and prevention performance was no longer significant. Bootstrapping confirmed a significant indirect association through OCSE, consistent with a full mediation pattern. **Conclusions**: Nurses’ workload appears to be indirectly associated with MDRPI prevention performance through OCSE. These findings suggest that strengthening nurses’ coping self-efficacy, alongside organizational strategies, may be essential for sustainable MDRPI prevention and patient safety improvement.

## 1. Introduction

### 1.1. Background

Medical Device-Related Pressure Injury (MDRPI) is an injury that occurs at the site of contact with a medical device applied for diagnostic or therapeutic purposes, characterized by localized tissue damage conforming to the shape of the device [1]. The 2019 National Pressure Injury Advisory Panel (NPIAP) international guidelines expanded the definition of MDRPI to include injuries caused by non-medical devices such as bed frames and household items, recommending that MDRPI be assessed and documented independently, distinct from general pressure injuries [2]. This expanded definition underscores that MDRPI is no longer a secondary complication but a distinct and increasingly prevalent patient safety concern across healthcare settings.

Indeed, recent studies report clinically significant MDRPI incidence rates in diverse clinical environments. Recent studies conducted across diverse clinical settings have consistently demonstrated that MDRPI is a prevalent patient safety issue worldwide [3,4], including in Korea [5,6,7]. In particular, evidence indicates that MDRPI incidence remains substantial across intensive care units and general wards, and that the risk increases with greater medical device use, underscoring the growing clinical relevance of MDRPI in modern healthcare environments [8,9]. Consequently, international guidelines classify all patients using medical devices as high-risk for MDRPI and emphasize the importance of structured nursing interventions, such as regular assessment of device contact areas, early detection of damage, application of preventive dressings, and pressure redistribution [2,10].

However, despite the existence of evidence-based recommendations for preventing MDRPI, the actual implementation of MDRPI prevention practices in clinical settings falls short of expectations. Previous literature indicates that Korean nurses’ MDRPI prevention performance levels are below average [5], and various organizational and environmental factors—such as excessive workload, priority conflicts, knowledge gaps, and staffing or resource constraints—impede consistent preventive practices [7,11]. Prior research demonstrating that appropriate staffing is essential for MDRPI prevention suggests that workload burden may be a primary factor associated with the task performance [12,13,14,15]. While positive links between workload and preventive nursing have been reported, previous research has largely been limited to descriptive or correlational associations, leaving the underlying mechanisms of MDRPI prevention performance unclear. Importantly, although workload, self-efficacy, and preventive nursing practices have each been examined in prior studies, empirical research explicitly investigating whether personal psychological resources statistically account for the association between workload and MDRPI prevention performance remains scarce. Therefore, grounded in Bandura’s Social Cognitive Theory (SCT), this study examines whether nurses’ occupational coping self-efficacy (OCSE) is statistically consistent with a mediating role in the relationship between workload and MDRPI prevention performance. By clarifying this mechanism, the study seeks to move beyond simplistic workload–performance models and to generate evidence that can inform more effective patient safety and quality improvement strategies in nursing practice.

### 1.2. Theoretical Framework

The theoretical basis of this study, SCT, is a representative framework that explains the formation and change in human behavior through interactions among personal, environmental, and behavioral factors [16]. The core concept of SCT, reciprocal determinism, emphasizes that individuals’ cognitive and emotional states (e.g., self-efficacy), their social and physical environment (e.g., workload), and their actual behaviors (e.g., performing MDRPI prevention) are not linked by unidirectional causality. Instead, they form a structure of mutual and cyclical influence [16,17].

In particular, self-efficacy, presented as the most central factor in SCT, refers to an individual’s belief in their ability to perform actions successfully in specific situations [18]. Among the dimensions of self-efficacy, OCSE is defined as an individual’s assessment of their ability to effectively manage clinical stressors, such as diverse demands, high workload, unpredictable patient conditions, and interpersonal burdens [19,20,21,22]. Within the SCT framework, OCSE represents a key personal factor through which environmental demands may be translated into behavioral responses. It is regarded as a crucial personal capacity for nurses, enabling complex clinical decision-making and sustained task performance [23]. Previous studies have also reported that nurses with high OCSE exhibit higher levels of preventive nursing practice and maintain task performance more efficiently while managing work stress [24,25]. Importantly, OCSE has been shown to attenuate the association between workload and work-related outcomes, suggesting a potential mediating role [26].

More sophisticated mechanisms—such as the process through which workload impedes MDRPI preventive practices and the role OCSE plays in this process—have not been sufficiently elucidated. In particular, empirical verification of whether the associations between workload and MDRPI prevention are purely direct or whether indirect pathways mediated by personal factors exist, as described by SCT, remains severely lacking. Accordingly, the present study aims to clarify the pattern of associations among workload, occupational coping self-efficacy, and MDRPI prevention performance, with a specific focus on the potential mediating role of OCSE within a statistical rather than causal framework. Based on SCT and prior empirical findings, the following hypotheses were formulated:

**Hypothesis 1 (H1).** 
*Nurses’ workload is negatively associated with MDRPI prevention performance.*

**Hypothesis 2 (H2).** 
*Nurses’ workload is negatively associated with OCSE.*

**Hypothesis 3 (H3).** 
*OCSE statistically mediates the association between nurses’ workload and MDRPI prevention performance.*

## 2. Methods

### 2.1. Study Design

This study employed a descriptive correlational design incorporating a mediation model to examine the indirect effect of OCSE on the relationship between workload and MDRPI prevention performance. Data were collected at a single time point, and the proposed mediation model was tested to explore potential mechanisms linking environmental and personal factors rather than to establish causal relationships. Given the cross-sectional nature of the data, the mediation analysis in this study was intended to examine patterns of statistical association rather than causal or temporal pathways. The model was conceptually grounded in SCT, which informed the selection of variables and hypothesized pathways among workload, OCSE, and MDRPI prevention performance.

### 2.2. Study Population

The participants were nurses working in the ICUs, general wards, and integrated nursing care wards of general hospital located in region P, South Korea, from May to July 2025. This study adopted a convenience sampling strategy based on institutional accessibility and feasibility, which is commonly used in exploratory mediation studies conducted in clinical settings. However, because participants were recruited from a single institution using convenience sampling, the potential for selection bias cannot be excluded. In particular, nurses who chose to participate may differ systematically from non-participants in terms of workload perception, coping resources, or interest in patient safety-related practices. The inclusion criteria were as follows: (1) registered nurses providing direct patient care and (2) at least six months of clinical experience. Only those who voluntarily provided informed consent were enrolled. Nurses working exclusively in administrative or managerial roles without direct patient-care responsibilities were excluded. Accordingly, the findings of this study should be interpreted with caution, and generalizability may be limited to nurses working in similar clinical settings and organizational contexts.

Sample size estimation was conducted using G*Power 3.1 based on the multiple regression model underlying the mediation analysis. Following methodological recommendations that mediation models require adequate power for detecting the smallest path in the regression sequence, we used the total-effect regression (workload → MDRPI prevention performance) as the most conservative criterion. A medium effect size (*f*^2^ = 0.15), α = 0.05, and power = 0.80 were assumed in accordance with Cohen’s guidelines and previous studies examining similar behavioral outcomes among nurses. A total of 12 covariates (workload, self-efficacy, gender, age, department, work type, marital status, household income, educational level, clinical experience, and average number of patients assigned per nurse), including demographic and work-related characteristics previously reported to be associated with preventive performance, were included to avoid model overestimation. Under these parameters, the minimum required sample size was 127. To account for incomplete or invalid responses, 195 nurses were initially recruited. After data screening, 14 responses were excluded due to predefined quality criteria, resulting in a final analytic sample of 181 participants, which exceeded the minimum required sample size and ensured sufficient statistical power for mediation analysis.

### 2.3. Data Collection

An online survey format was adopted to ensure accessibility for nurses working rotating shifts and to minimize disruption to clinical workflow. Because bedside nurses in general wards, ICUs, and integrated nursing care units frequently experience unpredictable workloads, an online platform allowed participation at a convenient time without interfering with patient care. The survey was administered through a secure, encrypted system approved by the hospital’s nursing administration, ensuring that all eligible nurses received equal opportunity to participate regardless of unit or work schedule. To ensure data quality, only one submission per device and IP address was allowed by the survey platform. All items were mandatory to minimize missing data; however, participants could withdraw before submitting their responses. Responses were screened for quality using predefined criteria. Cases were excluded if they demonstrated straight-lining patterns across multiple items, had an implausibly short completion time (<3 min), or had missing values due to early termination. Based on these criteria, 14 invalid responses were removed, resulting in a final analytic sample of 181 participants.

No personally identifiable information was collected. All survey data were stored on an encrypted, password-protected server accessible only to the research team, in accordance with institutional data protection policies.

### 2.4. Data Analysis

Data were analyzed using SPSS version 29.0 (IBM Corp., Armonk, NY, USA). The statistical significance level was set at *p* < 0.05 for two-tailed tests. Prior to conducting regression analyses, assumptions of normality, linearity, homoscedasticity, and independence of residuals were examined and met. Multicollinearity was assessed using variance inflation factors (VIF); all were below the commonly accepted threshold of 10, indicating no multicollinearity concerns.

Descriptive statistics, including frequencies and percentages for categorical variables and means and standard deviations for continuous variables, were used to summarize participants’ characteristics and major study variables. Cronbach’s *α* coefficients were calculated to assess the reliability of the measurement tools. Pearson correlation coefficients were computed to examine bivariate associations between workload, OCSE, and MDRPI prevention performance.

To examine the mediating effect of OCSE, a two-step analytical approach was employed. First, hierarchical multiple regression analyses were conducted to explore the relationships among variables in accordance with the conceptual framework proposed by Baron and Kenny [27]. This step was used to examine the direction and magnitude of associations among workload, OCSE, and MDRPI prevention performance rather than to formally confirm mediation. Control variables were selected based on their theoretical relevance within the Social Cognitive Theory framework, prior empirical evidence demonstrating associations with preventive nursing performance, and their potential role as confounding factors. Because all variables were measured concurrently, this analytical approach does not permit inference of temporal precedence or causal directionality among variables. Accordingly, the mediation model should be interpreted as a statistical representation of associations consistent with the theoretical framework, rather than evidence of a causal mechanism. Second, the statistical significance of the indirect effect was formally tested using the PROCESS macro for SPSS (v4.2, Model 4). Bootstrapping with 5000 resamples and bias-corrected 95% confidence intervals (CI) was applied, as this method does not rely on normality assumptions and is recommended for mediation analysis, particularly in cross-sectional designs. Mediation was considered statistically significant when the bootstrapped CI for the indirect effect did not include zero.

### 2.5. Measurement Tools

The instruments used in this study included measures for workload, performance in preventing MDRPIs, and OCSE. All instruments were selected based on prior evidence of validity and reliability in nursing populations and alignment with the theoretical framework of this study. Permission to use all instruments was obtained from the respective copyright holders or developers.

#### 2.5.1. Workload

Workload was measured using the Korean version of the NASA-Task Load Index (NASA-TLX) [28], which consists of six subdimensions: Mental Demand, Physical Demand, Temporal Demand, Performance, Effort, and Frustration. In the present study, the “Performance” subdimension was excluded from the workload score for both statistical and theoretical reasons.

First, confirmatory factor analysis showed that the Performance item had a factor loading below 0.50, indicating that it did not adequately represent the underlying construct of perceived workload. Second, prior research has noted that the Performance item differs conceptually from other NASA-TLX components because it reflects a self-evaluation of task outcome rather than the subjective burden experienced during task performance. Accordingly, several studies measuring perceived or subjective workload have excluded the Performance item to maintain construct purity [26,28,29].

After removing the Performance item, the remaining five items demonstrated satisfactory construct validity (factor loadings ≥ 0.50, AVE ≥ 0.50, CR ≥ 0.70). The total workload score was calculated as the mean of these five items, with higher scores indicating greater perceived workload. Accordingly, the total score reflects overall perceived workload across the remaining five demand-related dimensions rather than self-evaluated task performance. From a theoretical perspective, this modification shifts the focus of the workload construct from perceived task outcomes to the subjective cognitive and emotional demands experienced during task performance, which is consistent with the study’s emphasis on psychological coping processes. Methodologically, however, this modification means that the resulting workload score is not directly equivalent to the original six-item NASA-TLX composite. Therefore, direct comparisons with findings from studies using the full NASA-TLX should be interpreted with caution. In this study, the modified five-item scale showed excellent internal consistency (Cronbach’s α = 0.908).

#### 2.5.2. Medical Device-Related Pressure Injury Prevention Performance

The prevention performance tool used in this study was originally developed by Kim [5] as the Performance Scale of Preventive Activities for Medical Device-Related Pressure Injuries. This instrument consists of four domains: assessment (10 items), performance of preventive activities (3 items), records and reports (3 items), and education (1 item). Each item is rated on a four-point Likert scale ranging from 1 (not important at all) to 4 (very important). The total performance score was calculated as the sum of all items (range: 17–68), with higher scores indicating a higher level of nursing performance. In a previous study, Cronbach’s α was 0.93, and in this study, Cronbach’s *α* was 0.94.

#### 2.5.3. Occupational Coping Self-Efficacy

The Occupational Coping Self-Efficacy Scale for Nurses (OCSE-N) was adapted to Korean and used in this study [26]. This instrument consists of nine items divided into two subdomains: occupational burden and relational difficulty. Each item is measured on 5-point Likert scale ranging from 1 (strongly disagree) to 5 (strongly agree). Total OCSE-N scores were computed by summing the nine items (range: 9–45), with higher scores indicating higher OCSE. In a previous study, the Cronbach’s α for all items was reported as 0.89, with 0.79 for the occupational burden subdomain and 0.89 for the relational difficulty subdomain [26]. In this study, Cronbach’s α for all items was 0.89, and the reliability for the two subdomains was 0.79 for occupational burden and 0.89 for relational difficulty.

### 2.6. Ethical Considerations

This study was approved by the Institutional Review Board of Dongguk University WISE Campus (DGU IRB 20250008). The personal information of participants was kept strictly confidential, and participation was based on voluntary consent. Participants provided online informed consent after being informed of the purpose and procedures of the study, potential discomforts, the handling of personal information after study completion, and their right to withdraw at any time without disadvantage. The personal information of the research participants was kept strictly confidential and will be retained for three years after study completion before being destroyed.

## 3. Results

### 3.1. General Characteristics

A total of 181 nurses participated in this study. Most participants were female, and the sample was distributed across general wards, intensive care units, and nurse-managed integrated care wards. The majority worked rotating three-shift schedules and were unmarried. In terms of professional background, most nurses held a bachelor’s degree and had less than 10 years of clinical experience. Regarding workload context, participants most commonly cared for between 6 and 10 patients per shift. Detailed demographic and work-related characteristics are presented in Table 1.

### 3.2. Differences in Study Variables According to Participants’ Characteristics

Differences in workload, occupational coping self-efficacy, and MDRPI prevention performance according to participants’ demographic and work-related characteristics are summarized in Table 2. Workload did not significantly differ across demographic or work-related characteristics. In contrast, occupational coping self-efficacy differed according to selected characteristics. Higher levels of occupational coping self-efficacy were observed among older nurses, those working fixed day shifts, and married nurses. MDRPI prevention performance also varied by participant characteristics. Higher prevention performance was observed among married nurses, those with higher educational attainment, and nurses assigned a moderate number of patients per shift, whereas lower performance was noted among nurses with the highest patient assignments.

### 3.3. Correlations Among Study Variables

Pearson correlation analysis was conducted to examine the relationships among workload, occupational coping self-efficacy, and MDRPI prevention performance (Table 3). Workload was negatively correlated with both occupational coping self-efficacy and MDRPI prevention performance, whereas occupational coping self-efficacy was positively correlated with MDRPI prevention performance. Overall, the correlation patterns were consistent with the hypothesized relationships and supported the subsequent mediation analysis.

### 3.4. Mediating Effect of Occupational Coping Self-Efficacy on the Relationship Between Work Load and MDRPI Prevention Performance

**Figure 1:**
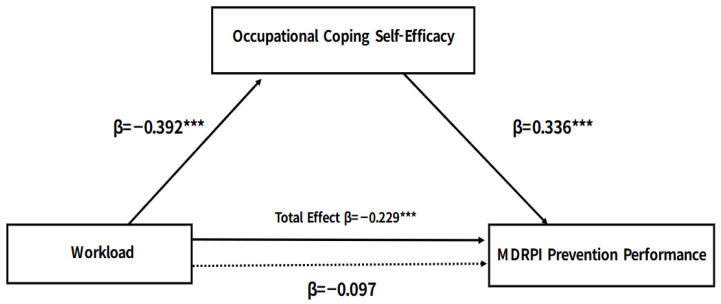
Conceptual mediation model showing the relationships among workload, occupational coping self-efficacy (OCSE), and MDRPI prevention performance. The model was adjusted for gender, age, work department, employment type, monthly income, education level, clinical experience, unit assigned per nurse, perceived MDRPI importance. *** *p* < 0.001.

The mediating role of occupational coping self-efficacy in the relationship between workload and MDRPI prevention performance is presented in Table 4 and Figure 1. Workload was significantly associated with occupational coping self-efficacy, and it was also associated with MDRPI prevention performance prior to the inclusion of the mediator. When occupational coping self-efficacy was included in the model, the direct association between workload and MDRPI prevention performance was no longer statistically significant, whereas occupational coping self-efficacy remained significantly associated with prevention performance. This pattern is consistent with a full mediation model, indicating that the association between workload and prevention performance was accounted for by occupational coping self-efficacy. Bootstrapping analysis further supported the mediation model, as the indirect effect of workload on MDRPI prevention performance through occupational coping self-efficacy was statistically significant, with the confidence interval not including zero.

## 4. Discussion

Based on SCT, this study examined the mediating role of OCSE in the relationship between nurses’ workload and MDRPI prevention performance. The results indicated that although workload did not have a significant direct association with MDRPI prevention performance, it exerted a significant indirect association through OCSE, which was statistically consistent with a full mediation pattern. These findings indicate that higher workload is not directly associated with poorer preventive performance but rather may be indirectly related by lowering nurses’ OCSE. Accordingly, these results should be interpreted as indicating a pattern of associations rather than evidence of a causal mechanism.

Therefore, these findings suggest that efforts to enhance MDRPI prevention may benefit from considering not only workload management but also interventions aimed at strengthening nurses’ occupational coping self-efficacy. This contrasts with previous literature suggesting a direct negative link between workload and practice implementation. Specifically, rather than a simple linear path in which high workload directly reduces performance, the findings suggest an interpretive pathway in which excessive workload may undermine OCSE, which in turn may be associated with a decline in MDRPI prevention behavior.

According to Bandura’s SCT, environmental (workload), personal (self-efficacy), and behavioral factors (preventive practices) interact cyclically within a structure of reciprocal determinism [16]. The findings of this study are consistent with this theoretical framework. Specifically, rather than environmental stressors such as workload directly affecting the behavioral factor of MDRPI prevention practices, the results suggest a more plausible indirect pathway in which they may be associated with these practices via the personal factor of OCSE. This demonstrates that even when nurses perform the same tasks in the same environment, the degree to which they perceive they can handle the work situation may meaningfully shape their actual behavioral performance. Previous studies have also reported that higher self-efficacy positively influences coping ability, resilience, and sustained preventive practices in stressful situations [30,31], which is consistent with the findings of this study. In interpreting these findings, it should be noted that workload was operationalized by excluding the NASA-TLX Performance subscale to focus on subjective cognitive and emotional demands. While this focus aligns with our psychological framework, the use of a modified scale requires caution when comparing these results with studies using the full instrument.

In the Korean clinical environment, the establishment of the Korean Association of Wound, Ostomy, and Continence Nurses (KAWOCN) in 2000 and the subsequent introduction of the WOCN system have led to the specialization of pressure injury management. Tasks previously handled by general nurses have increasingly shifted to WOCNs, who provide specialized care for chronic wounds, including assessment, dressing changes, education, and follow-up management for complications such as pressure ulcers, diabetic foot, and stomas [32]. However, according to the National Wound Care Strategy update (2023), the responsibility for pressure injury prevention and routine management lies with all clinical nurses, not solely with WOCNs [33]. The WOCN’s role is primarily to establish practice standards, provide education, and offer expert consultation [33,34,35]. While ward nurses are responsible for the daily processes of MDRPI prevention—such as verifying device placement, performing skin assessments, and applying prophylactic dressings [36]—a problematic perception persists. Some ward nurses misperceive MDRPI prevention as falling exclusively within the WOCN’s domain. Consequently, they tend to view it as having a lower priority than other nursing interventions, leading to missed preventive care and inconsistent implementation [5,37]. This observation is consistent with global trends indicating that role ambiguity often leads to the deprioritization of preventive care [38,39]. Collectively, these contextual factors partially explain our finding that simply reducing workload does not appear to be directly associated with improved prevention practices. This suggests that the barrier to implementation is not merely the quantity of work (workload) but also involves qualitative factors such as the meaning attributed to the task, role perception, and self-efficacy.

Furthermore, insufficient knowledge or underestimation of the importance of MDRPI prevention has been shown to hinder preventive practice [40]. Previous studies indicate that when nurses clearly understand the clinical significance of MDRPI and recognize their preventive role, their confidence in performing related tasks increases, which subsequently strengthens preventive behaviors [21,41,42]. Given that OCSE emerged as the key mediator within the statistical model used in this study, interventions must go beyond simple workload reduction and instead focus on systematically enhancing nurses’ knowledge and confidence. However, these practice implications should be interpreted with caution, as the cross-sectional design precludes conclusions about causal pathways or temporal ordering. Because MDRPI can develop rapidly and early assessment is critical, organizational support is essential to establish preventive practice as a core nursing competency.

Based on these findings, we recommend three practice-level strategies. First, self-efficacy–enhancing educational programs—including simulation-based training, skill rehearsal, and mastery experiences—should be incorporated into MDRPI prevention initiatives. Such interventions have been shown to improve both perceived capability and actual preventive behavior. Second, clarification of preventive roles and standardization of MDRPI protocols are essential, particularly in settings where WOCNs and general nurses share responsibilities. Clear task delineation and reliable practice guidelines can reduce cognitive burden and promote consistent preventive actions. Third, organizational reinforcement, including managerial feedback, real-time safety reminders, and adequate staffing to reduce competing cognitive demands, can strengthen nurses’ confidence and sustain preventive performance even under high workload conditions.

## 5. Conclusions

This study was conducted to identify the mediating role of OCSE in the relationship between nurses’ workload and MDRPI prevention performance. The analysis revealed that workload did not have a direct association with MDRPI prevention performance; however, a significant indirect association was observed through OCSE, which was consistent with a full mediation pattern. This indicates that, even within the same work environment, nurses’ perceptions of their ability to cope with work situations are a key factor associated with MDRPI prevention performance. Therefore, improving prevention performance may not be achieved solely through organizational interventions that adjust workload but rather a multidimensional approach that strengthens nurses’ individual capacities may be required. However, because this study employed a cross-sectional design, causal relationships and temporal ordering among workload, OCSE, and MDRPI prevention performance cannot be established. In addition, caution should be exercised when generalizing the findings because of the inclusion of nurses from a single medical institution in one region. Accordingly, the findings may not be generalizable to nurses working in other institutional or healthcare contexts. Furthermore, as all key variables were assessed using self-reported questionnaires, the possibility of common method bias cannot be ruled out. Although validated instruments were used and anonymity was ensured to reduce socially desirable responding, reliance on a single data source may have inflated the observed associations among workload, occupational coping self-efficacy, and MDRPI prevention performance. Future studies should adopt multi-center designs that include diverse hospital settings, longitudinal approaches, or causal and path analyses using structural equation modeling. Despite these limitations, this study provides a foundational basis for developing multifaceted interventions to enhance MDRPI prevention.

## Figures and Tables

**Table 1:** General characteristics of participants (N = 181).

Variables	Category	n	%
Gender	Male	20	11.0
	Female	161	89.0
Age (years)	≤25	49	27.1
	26~30	71	39.2
	31~35	36	19.9
	≥36	25	13.8
Work Department	Intensive Care Unit	59	32.6
	General Ward	66	36.5
	Nurse-Managed Care Integration Ward	56	30.9
Employment Type	Three-Shift Work	149	82.3
	Fixed Night Shift	8	4.4
	Fixed Day Shift	24	13.3
Marital Status	Single	130	71.8
	Married	51	28.2
Average Monthly Household Income (KRW)	Less than 2 million	1	0.6
	2 to 3 million	11	6.1
	3 to 4 million	106	58.6
	4 to 5 million	37	20.4
	5 million or more	26	14.4
Education Level	Associate Degree	42	23.2
	Bachelor’s Degree	138	76.2
	Master’s Degree or Higher	1	0.6
Clinical Experience (years)	6 months to less than 5	88	48.5
	5 to less than 10	53	29.3
	10 to less than 15	21	11.6
	15 to less than 20	14	7.7
	20 or more	5	2.8
Unit Assigned per Nurse (persons/duty)	≤5	58	32.0
	6~10	70	38.7
	11~15	38	21.0
	≥16	15	8.3

**Table 2:** Differences in study variables according to participants’ characteristic.

Variables		Workload		Occupational Coping Self-Efficacy			MDRPI Prevention Performance
		Mean ± SD	t or F (*p*)	Mean ± SD	t or F (*p*)	Mean ± SD	t or F (*p*)
Gender	Male	75.00 ± 13.24	−0.163 (0.871)	3.39 ± 0.55	1.379 (0.170)	3.73 ± 0.34	−0.503 (0.615)
	Female	75.66 ± 17.47		3.18 ± 0.64		3.79 ± 0.49	
Age (years)	≤25	78.35 ± 14.71	0.708 (0.548)	3.01 ± 0.57	5.198 (0.002) d > a	3.70 ± 0.49	1.719 (0.165)
	26~30	74.44 ± 18.93		3.23 ± 0.62		3.76 ± 0.47	
	31~35	75.81 ± 17.20		3.17 ± 0.54		3.83 ± 0.46	
	≥36	73.14 ± 15.47		3.60 ± 0.73		3.95 ± 0.46	
Work Department	Intensive Care Unit	73.86 ± 17.16	2.378 (0.096)	3.22 ± 0.56	0.023 (0.977)	3.75 ± 0.31	0.597 (0.554)
	General Ward	73.66 ± 19.20		3.20 ± 0.69		3.76 ± 0.52	
	Nurse-Managed Care Integration Ward	79.68 ± 13.35		3.20 ± 0.63		3.84 ± 0.55	
Employment Type	Three-Shift Work	75.67 ± 17.57	0.294 (0.746)	3.16 ± 0.60	3.541 (0.031) c > a,b	3.61 ± 0.41	0.540 (0.584)
	Fixed Night Shift	79.13 ± 8.04		3.10 ± 0.40		3.47 ± 0.32	
	Fixed Day Shift	73.88 ± 16.00		3.52 ± 0.80		3.56 ± 0.32	
Marital Status	Single	76.41 ± 16.64	1.038 (0.301)	3.11 ± 0.55	−3.561 (0.000)	3.72 ± 0.45	−2.756 (0.006)
	Married	73.49 ± 17.99		3.46 ± 0.74		3.93 ± 0.50	
Average Monthly Household Income (KRW)	Less than 2 million	82.00 ± 0.00	1.057 (0.380)	2.00 ± 0.00	2.022 (0.093)	3.00 ± 0.00	0.898 (0.466)
	2 to 3 million	82.00 ± 21.60		3.22 ± 0.96		3.64 ± 0.34	
	3 to 4 million	73.69 ± 17.10		3.18 ± 0.57		3.60 ± 0.43	
	4 to 5 million	78.63 ± 16.50		3.13 ± 0.53		3.54 ± 0.37	
	5 million or more	76.04 ± 15.22		3.44 ± 0.76		3.65 ± 0.34	
Education Level	Associate Degree	70.43 ± 20.05	2.585 (0.078)	3.15 ± 0.61	0.273 (0.761)	3.74 ± 0.42	3.682 (0.027)
	Bachelor’s Degree	77.18 ± 15.81		3.22 ± 0.64		3.55 ± 0.38	
	Master’s Degree or Higher	73.00 ± 0.00		3.00 ± 0.00		3.65 ± 0.00	
Clinical Experience (years)	6 months to less than 5	76.16 ± 15.67	0.251 (0.909)	3.11 ± 0.59	1.507 (0.202)	3.73 ± 0.47	0.573 (0.683)
	5 to less than 10	75.46 ± 20.78		3.21 ± 0.60		3.75 ± 0.49	
	10 to less than 15	75.05 ± 12.75		3.36 ± 0.66		3.88 ± 0.37	
	15 to less than 20	71.93 ± 18.55		3.48 ± 0.62		3.84 ± 0.41	
	20 or more	79.40 ± 9.61		3.36 ± 1.27		3.78 ± 0.28	
Unit Assigned per Nurse (persons/duty)	≤5	77.58 ± 13.73	1.494 (0.218)	3.21 ± 0.51	1.684 (0.172)	3.79 ± 0.33	3.225 (0.024) c > d
	6~10	76.61 ± 17.90		3.13 ± 0.60		3.71 ± 0.52	
	11~15	70.50 ± 20.24		3.39 ± 0.80		3.97 ± 0.44	
	≥16	76.00 ± 14.40		3.09 ± 0.69		3.63 ± 0.66	

**Table 3:** Correlations among study variables.

Variables	1	2	3
1. Workload	1		
2. Occupational coping self-efficacy	−0.380 **	1	
3. MDRPI prevention performance	−0.235 **	0.397 **	1

** *p* < 0.01.

**Table 4:** Mediating effect of occupational coping self-efficacy in the relationship between workload and MDRPI prevention performance.

	Pathway	B	SE	β	t	*p*	R^2^	F (*p*)	95% CI
1	Workload → Occupational Coping Self-Efficacy	−0.014	0.003	−0.392	−5.563	<0.001	0.209	4.496	−0.020~−0.008
2	Workload → MDRPI Prevention Performance	−0.006	0.002	−0.229	−3.117	<0.001	0.139	2.755	−0.010~−0.002
3	Workload → MDRPI Prevention Performance	−0.003	0.002	−0.097	−1.284	0.201	0.229	4.555 (<0.001)	−0.007~0.001
	Coping Self-Efficacy → MDRPI Prevention Performance	0.252	0.057	0.336	4.422	0.000			0.140~0.364
		**Indirect effect**			**Boot SE**			**95% CI**	
	Workload → Occupational Coping Self-Efficacy → MDRPI Prevention Performance	−0.004			0.001			−0.006~−0.002	

## Data Availability

The raw data supporting the conclusions of this article will be made available by the authors on request.
